# Towards Improved Eye Movement Biometrics: Investigating New Features with Neural Networks

**DOI:** 10.3390/s25144304

**Published:** 2025-07-10

**Authors:** Katarzyna Harezlak, Ewa Pluciennik

**Affiliations:** Department of Applied Informatics, Silesian University of Technology, Akademicka 16, 44-100 Gliwice, Poland

**Keywords:** eye movement, feature selection, classification, biometrics, neural network, LSTM

## Abstract

Providing protected access to many everyday-used resources is becoming increasingly necessary. Research on applying eye movement for this purpose has been conducted for many years. However, due to technological advancements and the lack of stable solutions, subsequent explorations remain valid. The presented work is one of such studies. Two methods of biometric identification based on eye movements that utilize neural networks have been developed. In the first case, a feature vector was constructed from a 100-element time series depicting eye movement dynamics, which included velocity, acceleration, jerk, their point-to-point percentage changes, and frequency-domain representations. The same eye movement dynamic features were used in the second method, but this time, statistical values were calculated based on the previously defined time series. Long Short-Term Memory (LSTM) and dense networks were used in the user identification task in the first and second approaches, respectively. In the exploration, the publicly available GazeBase dataset was used, from which data collected for the ‘jumping point’ stimulus were chosen. The obtained results are very promising, with an accuracy of 96% for the LSTM model and the time series feature vector set and 76% for the second method. They were achieved over a three-year time span of eye movement recordings; however, different time periods were investigated, as well as various numbers of stimulus positions.

## 1. Introduction

One of the most crucial issues in the current world is preventing unauthorized access, protecting against cyberattacks, and ensuring the confidentiality, integrity, and availability of data. As a result, we can observe the rapid growth of cybersecurity—a field dedicated to implementing a range of practices, technologies, and procedures ensuring data safety. A key aspect of cybersecurity is access control, which involves determining who can access system resources and under which conditions. Central to this process are identification and authorization mechanisms.

Identification refers to recognizing the identity of a user, device, or system. However, identification alone does not guarantee access; it must be followed by authentication, which confirms the claimed identity. Identification and authentication typically rely on one or more factors: something the user knows, such as a password; something the user possesses, like a security token; or something inherent to the user, such as biometric characteristics [1].

Biometric identification offers several advantages: it eliminates the need to remember passwords or carry physical tokens, and biometric traits cannot be easily forgotten or misplaced. Commonly used biometric identifiers include fingerprints, facial features, retina patterns, and iris scans. However, these physical characteristics are vulnerable to replication using modern technologies. For instance, fingerprints can be lifted from surfaces like glass, and high-resolution devices can capture iris patterns. Even voice characteristics can now be convincingly mimicked. To address these vulnerabilities, researchers are increasingly focusing on behavioral biometrics, which are less susceptible to such threats. Examples include typing patterns, gait, mouse movement, blood volume changes, and eye movements—the latter being the focus of this study.

Eye movements are voluntary or involuntary shifts of the eye that help gather visual information. Two primary types of eye movement events are:

- Fixations—when the gaze remains relatively stable on a single point, allowing the brain to process visual input,
- Saccades—rapid movements between fixation points, during which no visual information is acquired.

Fixations typically last between 200 and 300 ms, depending on the visual task, and include micro-movements such as drifts, microsaccades, and tremors, which help maintain visual acuity. Saccades are characterized by their amplitude (ranging from 4 to $20^{\circ }$), speed (300–$500^{\circ }$/s), and duration (a few tens of milliseconds). Additionally, saccadic latency can occur, which is the delay between a stimulus position change and the eye’s response, usually ranging from 150 to 200 ms [2,3].

The premise of using eye movements for biometric identification lies in the assumption that these patterns contain unique, individual-specific features that remain stable over time. While preliminary research has explored this potential, a standardized solution has yet to emerge. This gap motivated the present study, which aims to define a robust set of eye movement features and develop a processing method capable of reliably identifying individuals.

The presented research contributions are:

- elaborating new feature sets and methods for eye-movement-based biometric identification,
- evaluation and comparative assessment of the proposed methods based on their classification performance,
- investigate the effectiveness of the developed solutions in terms of time span and the number of stimulus points.

## 2. Related Work

Eye movement biometrics have been investigated for over two decades [4,5], based on the hypothesis that individual patterns of eye movement are sufficiently distinctive to enable personal identification. Experiments in this field typically focus on two main components: the physiological, which pertains to the mechanics of the oculomotor system, and the behavioral, which is associated with neural processes governing gaze control. These components provide a broad foundation for research.

A widely adopted experimental paradigm involves eliciting controlled eye movements, as physiological responses are generally more straightforward to quantify and reproduce. A typical example is the usage of the “jumping point” stimulus, wherein participants are instructed to follow a point that periodically shifts position on a screen [4,6,7]. This type of stimulus minimizes voluntary decision-making because the gaze direction is externally guided.

Early contributions to eye movement-based biometric identification were introduced by Kasprowski and Ober [4], who employed a jumping point paradigm and extracted cepstral coefficients from binocular eye movement signals. Their approach, evaluated using several classifiers: NB (Naive Bayes), DT (Decision Tree), kNN (k-Nearest Neighbor), and SVM (Support Vector Machine), achieved the best performance with kNN (k=3), yielding a FAR (False Acceptance Rate) of 1.48% and FRR (False Rejection Rate) of 22.59%. Bednarik et al. [8] extended this line of research by utilizing a moving cross stimulus and incorporating features such as pupil diameter, corneal reflection distance, and gaze velocity for 12 participants. Their method, limited to a single session, achieved classification accuracies ranging from 42% to 92%. Zhang et al. [9] also investigated saccadic eye movements for jumping point stimulus in the user verification task across 109 participants. The feature set included amplitude, latency, angular velocity, and acceleration metrics. The classification was performed using MLP (Multi-Layer Perceptron), RBF (Radial Basis Function), SVM, and LDA (Linear Discriminant Analysis), yielding accuracies ranging from 63% to 85%. A broader comparative analysis was presented by Galdi et al. [10], who reported identification accuracies ranging from 58% to 79% for the conducted explorations under varying experimental conditions. George and Routray [11] proposed a score-level fusion framework based on fixation and saccade features extracted from eye-tracking data and processed using Gaussian RBF networks. Their method, evaluated on the BioEye 2015 dataset, achieved a Rank-1 accuracy of 92.38% in short-term tests. Harezlak and Blasiak [1] introduced, in addition to statistical values of velocity and acceleration, a frequency-domain approach, along with the Largest Lyapunov Exponent, using RF (Random Forest) classifiers. They achieved 100% accuracy; however, for a mixed dataset from two sessions of eye movement recording.

Between 2012 and 2015, three benchmark competitions [7,12,13] were organized to evaluate biometric systems under diverse stimuli, including jumping points, face observation, and text reading. These studies highlighted the challenges posed by template aging and reported accuracies between 40% and 60%. Bayat et al. [14] employed a combination of fixation, saccade, and pupil response features during reading tasks for 40 experiment participants. Using classifier ensembles (MLP, RF, and LMT (Logistic Model Tree)), they achieved a Rank-1 accuracy of 95.31% and an average EER of 2.03%. Other studies have explored static image viewing [5,15,16] and dynamic stimuli [17,18], with face perception frequently used due to its individualized gaze patterns.

Liao et al. [19] conducted real-world experiments using wearable eye trackers during wayfinding tasks. Data were collected for 39 subjects. The proposed system, evaluated using RF and multiple feature sets, achieved 78% identification and 89% verification accuracy with a 10-fold cross-validation and the LORO (Leave-One-Route-Out) solution. Seha et al. [20] assessed low-frame-rate devices for driver authentication (55 participants). They reported 96.67% Rank-1 accuracy for multimodal input and 93.94% for eye blinking alone. D’Amelio et al. [21] introduced a novel approach based on foraging theory, modeling gaze behavior as a stochastic process. Their method, evaluated on the FIFA dataset (https://github.com/keisuke198619/SoccerTrack, accessed on 8 July 2025), achieved 93.75% accuracy and 4.6% EER for 8 subjects. Recently, Ozdel et al. [22] analyzed LFI-based eye movement data across diverse tasks (talking, reading, problem-solving, watching a video, typing, walking, and cycling), achieving 93.14% accuracy and 2.52% EER using SVM and LightGBM classifiers. They utilized a dataset presented in [23], which contained data for 10 participants and was used for human activity recognition.

Latest advancements in deep learning have led to the adoption of CNNs (Convolutional Neural Networks) and LSTMs (Long Short-Term Memory) for biometric identification. Prasse et al. [24] utilized a CNN model developed for processing micro- and macro-eye movements, described in Makowski et al. [25]. They reported an accuracy ranging from 34% to 80%, depending on the stimulus and the device used for data recording. Four data sets were utilized, with participant numbers ranging from 22 to 173. The same model was used in [26] to detect presentation attacks in an experiment where a jumping point with various display times was employed. This model achieved EERs of less than 10% with usage of JuDo1000 dataset (https://osf.io/5zpvk/, accessed on 8 July 2025) containing recordings for 150 subjects.

Lohr et al. [27] employed a DenseNet model validated on text-based stimuli for 59 participants, reporting EERs between 0.5% and 10%. Yin et al. [28] utilized the GazeBase FXS subset [29], focusing on fixation and saccade detection. The experiments were also conducted for jumping point (RAN), horizontal saccades (HSS), and text reading (TEX) parts of GazeBase. They extracted motion information and saccade distribution maps, which were processed using a dual-branch CNN architecture. Their experiments focused on authentication tasks and were conducted on both closed and open datasets, yielding EERs of 5.25% and 10.62%, respectively. The data from 290 subjects were used for training and validating the model, and the data from 31 subjects were used for testing.

LSTM-based models have also demonstrated strong performance. Wang et al. [30] used bidirectional LSTM networks on reading data, achieving 86.5% Rank-1 accuracy and 19.4% EER for the Provo Corpus dataset (https://osf.io/sjefs/, accessed on 8 July 2025), which includes data for 84 native English speakers reading 55 text samples. The authors in [31] proposed a hybrid model combining transformers, attention-enhanced LSTMs, and frequency-domain self-attention. Experiments on three datasets with varied stimuli yielded EERs ranging from 0.06% to 0.22% (depending on the time span), with each stimulus evaluated independently. The first set contained samples gathered by the authors for 203 subjects using the jumping point with two consecutive sessions. The second and third ones were GazeBaze (RAN, TEX, FXS, HSS) and JuDo1000. The work [32] describes GazeNet, a deep learning-based biometric recognition framework that leverages short-term, raw eye movement data processed through a three-layer LSTM network. Forty participants’ task was to judge whether the two molecular models (either 2D dash-and-wedge or 3D ball-and-stick) were the same or different. GazeNet achieved a Rank-1 accuracy of 96.3% and an EER of 0.85%.

It should be noted that raw eye movement data (such as gaze positions) are always processed to extract features suitable to train classifiers. Some studies rely on basic statistical measures [33,34,35], while others employ more advanced techniques, such as comparing the distributions of fixation and saccade characteristics [33]. In addition, ref. [6] presents an attempt to model the oculomotor plant mathematically using eye movement data. Other approaches apply signal processing techniques, including Fourier, wavelet, and cepstral transforms, to analyze the temporal dynamics of eye movements [4,8,36]. Spatial analysis methods are also used, such as generating and interpreting heat maps or scan paths based on gaze position data [15,37]. Some approaches apply a fusion of various feature types [14,19,20,28,37].

The reviewed studies demonstrate a wide range of strategies in eye movement biometrics, often combining various stimuli, features, and classification techniques to enhance identification performance. However, there remains potential for further research aimed at simplifying the identification process, particularly by optimizing feature selection and model complexity while maintaining high accuracy. Importantly, the quality, diversity, and size of the datasets used for training are critical to the success of machine learning models. In the case of eye movement biometrics or generally behavioral biometrics, the long-term stability of identification should be carefully investigated.

## 3. Materials and Methods

In the development of neural network-based learning models, both the network architecture and the training data play pivotal roles. While the network’s topology determines the computational demands during training and inference, the training dataset fundamentally shapes the model’s ability to generalize and perform effectively. A high-quality, representative dataset is essential, as it directly influences the model’s capacity to capture the underlying patterns and details of the problem domain.

### 3.1. Eye Movement Dataset

The dataset employed in this study, known as GazeBase, originates from [29]. GazeBase comprises monocular (left eye) movement recordings collected from 322 college-aged participants across seven different tasks, one of which involved a randomly jumping point. Data collection spanned 37 months, utilizing the EyeLink 1000 eye tracker (SR Research, Ottawa, ON, Canada) with a sampling rate of 1000 Hz, across nine sessions (each comprising two trials, with or without a break). Fourteen individuals from the original group of 322 took part in every session.

During the experiments, participants were seated with their heads stabilized at a distance of 550 mm from a monitor with a resolution of 1680×1050 pixels (physical dimensions: 474×297 mm). The experiments were conducted in a quiet laboratory with uniform background lighting to ensure consistent visual conditions. Initially, both gaze and stimulus positions were recorded in pixel coordinates and later converted into DVA (degrees of the visual angle). Table 1 presents details on each recording session.

### 3.2. Eye Movement Feature Extraction

As mentioned, the data used in the experiments were recorded at a sampling rate of 1000 Hz, and a point in one location was displayed for 1 s. There were 100 such locations. Therefore, 1000 recordings were obtained for each point position. For the purpose of this study, they were divided into *n*-element segments. An assumed target segment size *n* was set to 100 ms. Of the 10 segments, only G (G = 3) was considered in further studies. They included three eye movement events: saccadic latency, saccade, and fixation. However, due to the feature extraction process, the initial size of the selected eye movement signal was set to 303 to ensure enough recordings for calculating velocity, acceleration, and jerk. Subsequently, for each dynamic feature, three 100-element time series were extracted from the obtained subsets.

Two groups of features were defined from each segment. The first one represents seven time series, including the first three derivatives of horizontal eye position—point-to-point: (1) velocity, (2) acceleration, and (3) jerk, their corresponding point-to-point percentage changes and the amplitude spectrum—100-element time series representing the intensity of the different frequencies present in a signal, defined based on eye-movement velocity Fourier transform (Ft). After extracting these features, a detailed description of which is provided in Table 2, all time series consisted of 100 elements.

Before calculating features, some preprocessing steps were undertaken. The GazeBase dataset contains missing samples denoted by non-zero values in the validation field and NaN values for corresponding gaze positions. The missing sample occurrence is caused by the inability to correctly read the gaze position (due to blinks or partial occlusions of the eye). Recordings for stimulus positions that included NaN values were removed before further analysis. As a result, out of 100 stimulus locations, 50 were selected for use in the experiments. All time series obtained from the remaining recording subsets were scaled using the min-max scaling technique. The data scaling method was applied to both the training and test sets.

The second feature group consisted of statistical measures—min, max, mean, and std—calculated for four out of seven elements from the first feature group. They were velocity, acceleration, jerk, and the Fourier transform amplitude spectrum. It gave a 16-element set for each segment.

**Figure 1:**
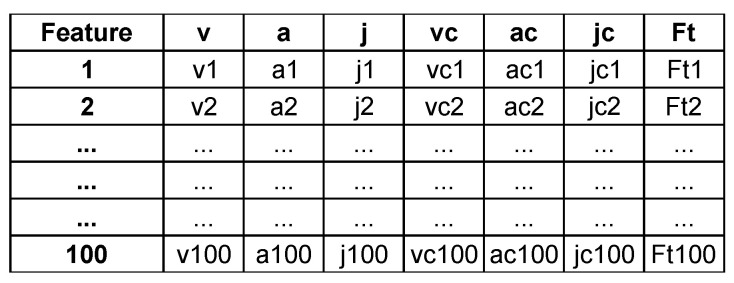
Feature vector based on seven time series for one segment.

**Figure 2:**
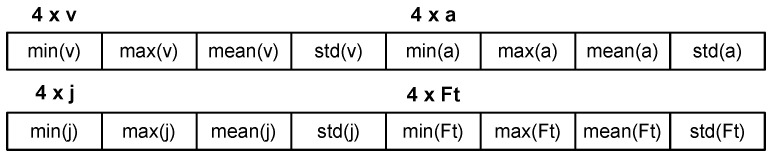
Feature vector based on sixteen statistical values for one segment.

The aforementioned features constituted two types of feature vectors and are presented in Figure 1 and Figure 2.

Taking into account that:

- *U* users took part in an experiment,
- during one session Si, the stimulus was presented in *X* positions,
- *G* segments were defined for each stimulus position,

the total number of samples for one person equaled:

- FVn=G×X for one session Si
- T_FVn=S×FVn for all sessions *S*.

### 3.3. Classification Methods

The previously defined groups of features were applied in two eye-movement-based identification tasks, for which two neural networks were developed. A recurrent LSTM with feedback connections, which is well-suited for processing time series data of varying lengths. Additionally, it is capable of capturing temporal dependencies (patterns) and can remember important information for a long time. The network consisted of two recurrent layers, one dense layer, and an output layer. All but the last had a number of units *u* = 64, while the unit number for the last one was dependent on the number of labels *l* in the dataset used. Between the LSTM layers, a dropout layer was used with a rate of 0.5.

**Figure 3:**
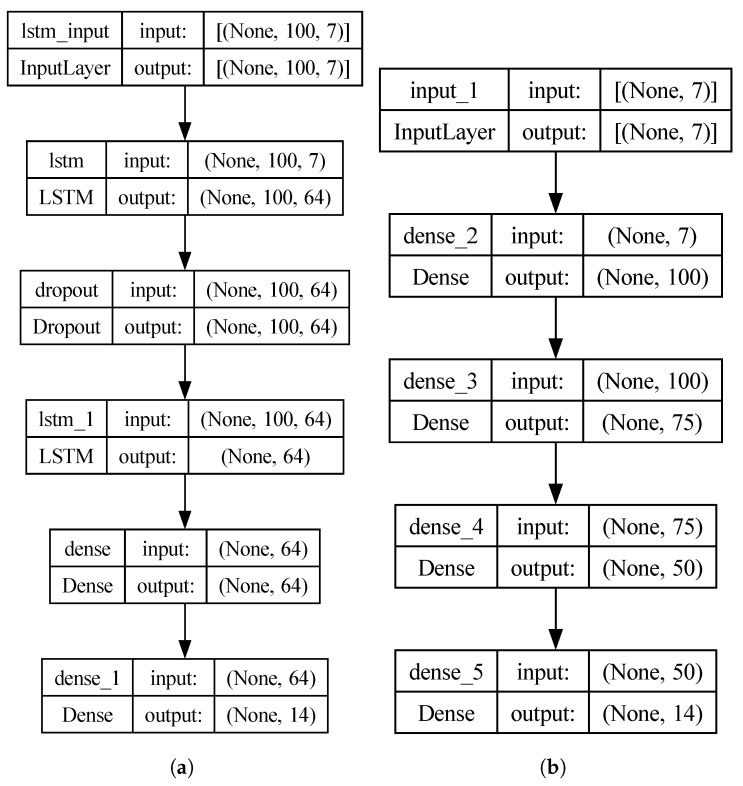
The models used in the experiments. (**a**) the LSTM model (**b**) the Dense one. The number of labels, equal to 14, was presented for the experiment, which utilized all nine sessions.

The second network was a dense neural network with three hidden layers consisting of *n* neurons equal to 100, 75, and 50, respectively, and an output layer with *l* neurons. Such architectures were selected based on preliminary studies and are presented in Figure 3—(a) the LSTM model and (b) the Dense one.

Both networks were compiled with the Adam optimizer [38] with standard parameters (learning rate equal to 0.001) and the categorical crossentropy loss function.

### 3.4. The Analyzed Scenarios

The primary objective of the research was to evaluate the effectiveness of the proposed feature sets in eye-movement-based biometric identification over the long-possible period. Therefore, the first scenario assumed the usage of sessions from the GazeBase dataset, which were obtained within the most extended break between the first and last session. There were *S* = 9 such sessions conducted within the three-year time period, each including two trials. Because the different rules were applied for the second trial participation, only the first trial from each session was taken into account to ensure a comparable set of samples for the method validation. They involved 14 participants, giving *l* = 14 labels. As mentioned, out of 100 stimulus positions, 50 were chosen as *X* value, which resulted from the necessity of removing NaN values.

The structure of the dataset also provided an opportunity to apply other scenarios that verified the elaborated set of features under different conditions. Therefore, additional experiments were conducted to ascertain the influence of:

- the number of stimulus positions
- and the number of sessions

used for model training on its performance. It must be emphasized that with a reduction in the number of sessions, more participants become available, causing an increase in the number of labels *l*.

### 3.5. Training, Testing and Evaluation Phase

The above-described scenarios were realized using the cross-validation technique, specifically the Leave-One-Session-Out (LOSO) method. Given *S* sessions for an experiment, the model was trained and validated *S* times. In each iteration, S−1 sessions were utilized for training, and the remaining one was used for model validation. The process was repeated as many times as the number of sessions used in the experiment. The session applied for testing varied in each run.

To evaluate the performance of the proposed models, several metrics were computed. They included the Accuracy, F1 Score, and Cohen’s Kappa. However, because for one user FVn samples were available during the testing phase, each sample was initially classified independently, providing probability distribution vectors $P_{i}$ for every possible class (user identity) *l*. Therefore, given probability vectors for *X* point positions and *G* segments, the probability vector for the user $U_{i}$ was calculated as

(1) $$P_{U_{i}}=\frac{\sum_{j}^{X\times G}P_{i_{j}}}{X\times G}$$

Finally, a user class $U_{l}$ was determined as the label *l* with the highest probability:

(2) $$U_{l}=argmax_{P_{U_{i}}}\left(P_{U_{i}},l\right)$$

## 4. Results

All tests, the results of which are presented in this section, were conducted using a computer equipped with an Intel(R) Core(TM) i7-14700KF 3.40 GHz processor, Dalian, China, 32.0 GB of RAM, an NVIDIA GeForce RTX 4070 GPU with 12 GB of RAM, Santa Clara, CA, USA, and a 64-bit Windows operating system. The experiments conducted with time-series-based feature sets utilized the model presented in Figure 3a, while in the second group of tests, it was the model visible in Figure 3b. Preliminary experiments were conducted to determine the number of epochs for training neural networks. These experiments revealed that 150 epochs were sufficient to achieve satisfactory performance. The early stopping parameter was omitted to ensure comparable conditions for comparing results obtained from different scenarios.

### 4.1. Verification of the Time Series-Based Feature Set in the Biometrics Task with Changing Number of Stimulus Positions

As mentioned, the first analyzed scenario aimed to verify the proposed feature set in biometric identification over a long-possible period; in the case of the dataset used, this was a three-year term. This dataset consisted of 9 sessions during which 14 participants were involved. Therefore, the total number of samples T_FVn in a feature set was 9×14×X, where *X* represents the number of stimulus positions chosen for the experiment purpose. Three cases were considered for X={50,35,25} to investigate the influence of the stimulus position number on model performance. The T_FVn values for each *X* are presented in Table 3. The table also shows the number of samples used for training and testing the model.

Because the LOSO cross-validation was used, the model was trained and validated *S* times. Therefore, the calculated metrics—Accuracy, F1 Score, and Cohen’s Kappa—were averaged over all runs. Their values are provided in Table 4, with standard deviation shown in brackets. As can be observed and as was expected, the decreasing number of samples affected the model’s performance. However, it remained at a reasonable level. While for 50 stimulus positions, the accuracy was, on average, 96%, for 35 and 25 positions, it was 90% and 79%, respectively. Moreover, examining the results for 50 and 35 positions, it can be observed that, considering the standard deviation, their ranges overlap. What can also be noticed is an increasing standard deviation for all metrics, which may result from a decrease in the number of stimulus positions, causing a lower number of samples for model training. With a small set of samples, the standard deviation may be less stable and more susceptible to extreme values.

### 4.2. Validation of the Model’s Efficiency for Varying Numbers of Sessions Using Time-Series-Based Feature Sets

In the second considered scenario, the proposed model and time-series-based feature sets were applied in several experiments that differed in the number of sessions used in cross-validation model evaluation. There were six such experiments, including from 8 to 3 sessions. The time span between:

- the first and the eighth session was approximately two and a half years,
- the first and the seventh session was approximately two years,
- the first and the sixth session was approximately one and a half years,
- the first and the fifth session was approximately one year,
- the first and the fourth session was approximately seven months,
- the first and the third session was approximately six months.

Detailed information regarding specific sessions is available in Table 1 in Section 3.1.

As mentioned before, with a decreasing number of sessions, a larger number of participants became available. It provided the possibility to verify the methods for extended sets of labels, sometimes with eye movements recorded at a similar session-break interval.

To ensure the same stimulus configuration as in the previously performed tests, only participants who attended all analyzed sessions were included in a particular experiment. Additionally, participants whose eye movement recordings included too many NaN values to ensure enough (*X* = 50) stimulus positions were also excluded from further explorations. The final label number for each experiment is given in Table 5. This table includes the number of samples used in experiments as well as those utilized in training and testing models.

The metric values obtained in each experiment are shown in Table 6. The outcomes revealed worse model performance in all analyzed cases compared to those achieved in the first analyzed scenario, despite the larger amount of data used for training. It is caused by the decreasing number of samples available per participant.

In the first scenario, with 9 sessions involving 14 participants, 1200 samples from the training set were available for each user. For the 8 and 7 sessions, the numbers were 1050 and 900, respectively. The decreasing pattern was maintained for the other experiments. When 6 and 5 sessions were used, 750 samples were attributed to a single person; for 4 sessions, this number was 600, and for 3 sessions, each participant was associated with 450 samples.

In scenarios using 8 and 7 sessions, the number of labels *l* was approximately twice as big as in the 9-session scenario. As a result, a larger number of samples was used for training the model; however, the data growth rate is not the same as that of the labels because fewer sessions are available. A higher number of classes makes the classification task more challenging, which results in reduced model accuracy. Nevertheless, these metrics still assume, on average, high values of 87% for the 8-session experiment and 83% for the 7-session one. As in the earlier case, the analysis of these outcomes in consideration of the standard deviation reveals that their ranges overlap. Good performance is also observed when six sessions are used in tests. The effectiveness is lower compared to previous cases, with an accuracy of 73%. However, the number of participants was 3 times higher in this scenario.

The influence of the number of labels *l* on the model’s performance can be easily seen when comparing the number of participants and the number of samples used to train the model in 5- and 6-session experiments: the same model and the same amount of data for its training yield different validation outcomes. The results from experiments with three and four sessions also confirmed that for a higher number of participants, more data is needed to develop an effective model.

### 4.3. Experiments with the Usage of Statistic-Based Feature Vectors

The second group of features, based on statistical values characterizing eye movement in particular segments, was used similarly to the previously described experimental scenarios. For their purpose, the second model was applied (see Figure 3b). As shown in Table 7, the model trained with this feature set type was less efficient than the one defined based on time series. The difference for the best results is approximately at the 20% level: previously obtained 96% compared to 74% in this part of the tests.

The decrease in the number of stimulus points from 50 to 35 and 25 revealed a further reduction in model performance. When the results for 35 and 25 stimulus positions are examined, it becomes apparent that they overlap when the standard deviation is taken into account. Nonetheless, the accuracy stayed above the chance level. However, when the number of sessions was limited, which was associated with an increase in the number of participants, the model’s efficiency dropped below this level. The outcomes from Table 8 show that for such a constructed feature vector and a larger set of classes, a bigger number of samples is required to make the identification process efficient.

## 5. Discussion

The presented research aimed to introduce a novel solution for biometric identification based on eye movement signals, which included defining a feature vector and selecting a model that would effectively perform the classification task. Two types of feature vectors were built. One was represented by several time series defined based on characteristics of eye movement dynamics in chosen signal segments. The feature vector with such components was used for the first time in this research.

To define the second vector type, some statistics were calculated for several time series from the first set. Although a similar approach was used in [1], it differed in the way the feature vector was constructed and the eye movement features employed.

For each feature set, a model was defined, which was used in various classification experiments. The developed time-series-based method proved robust, achieving an accuracy of 0.96 and F1 scores of up to 0.95. These results pertain to eye movement recordings collected over a three-year period, indicating the effectiveness of the method in the long term. The very good performance was also preserved when the number of stimulus positions was reduced, giving an accuracy of 0.90 (0.79) and F1 scores of 0.87 (0.73) for 35 (25) points. These satisfactory outcomes were achieved for 14 participants over nine sessions. The model consistently exhibits high performance when eight or seven sessions are considered, with double the number of participants and the data collection period still exceeding two years. Its accuracy remains at 87% and 83% levels, respectively.

For all experiments, execution time was also collected. The training time for the models varied depending on the number of data points and participants included in the experiments. For example, the LSTM model trained on data from 50 points and 14 participants across 9 sessions required a total of 5411 s (approximately 90 min), with an average of 601.22 s per LOSO iteration (approximately 10 min). The longest training time was observed for a model using 50 points from 59 participants across 6 sessions, totalling 8407 s (approximately 140 min), with an average of 1401.17 s per iteration (approximately 23 min). Other configurations with 8, 7, 5, 4, and 3 sessions required 7988, 6456, 7275, 6182, and 3414 s in total, respectively, corresponding to average iteration times of 998.5, 922.29, 1455.00, 1545.50, and 138.00 s. In contrast, flat models were trained significantly faster—approximately 8 times quicker—requiring between 394 and 1083 s in total, with average iteration times ranging from 78.11 to 186.00 s.

Promising results were also obtained in previously conducted works, for example, in [9]. The reported classification efficiency was 83% for 109 users, which is a higher label number than in this study case; however, measurements were recorded from every subject during a single session. The high classification performance was achieved in [11] using part of the dataset employed in the current research. The Rank-1 accuracy of 90.10% and 92.38% was achieved for 153 users with ‘jumping point’ and text stimuli, respectively. However, once again, such outcomes were obtained for two sessions organized 20 min apart. When sessions with a one-year interval and 37 users were considered, the Rank-1 accuracy dropped to 79.31% and 83.41%. It should be additionally emphasized that a more extended feature vector and a more complex network were used compared to the one presented in this paper.

There are also other works that reveal very good outcomes, yet obtained based on one-day recording sessions [14,19,20,21,22]. The method elaborated on such datasets did not account for possible changes in eye movement characteristics over a longer time range, which is an important factor that makes the identification process easier. There were four sessions used in [24] with a temporal lag of at least one week between any two sessions. These data were used to compare the effects of temporal and spatial resolution on classification results. However, biometric identification, achieving an accuracy of 80%, was performed for the dataset collected within a single session for 32 participants.

The same eye movement dataset as used in this research, with an extended period of data recording, was applied in [27,28]. Both papers, however, focused on the authentication process and considered open- and closed-set scenarios. As a model, CNN networks were used, and for their training, eye movement data registered for various stimuli was utilized in both works. The models’ performance was presented using the EER metric, and satisfactory results were reported in both papers. Nonetheless, because different tasks were addressed and different metrics applied, a direct comparison of the results achieved in those studies and those presented in this paper is difficult. The primary difference that can be noted is the simpler method employed in the currently presented research, which is based on a single stimulus type and involves fewer data preprocessing steps.

The next study, worth mentioning, is work [31], in which, among others, the same dataset was utilized. The authors reported their results using the EER metric. The value for this metric suggests that for Round 1 (EER = 0.08), the EmMixformer identified users with an accuracy of 92% in the best case. However, this outcome was achieved using data that came from the same eye movement recording session. Moreover, this result was obtained using a much more complex solution than the one proposed in this research.

The comparable studies with the best-achieved results are collocated in Table 9. Based on the presented results, it can be stated that the solutions developed in this research, although simpler in construction, outperform those previously proposed.

The second of the developed methods, utilizing statistical values of some time-series-based features, revealed a weaker model’s efficiency. This approach is promising, with an accuracy of 0.74 and an F1 score of 0.67, but only for the most extensive period of eye movement recordings involving 14 participants. Although it compares favorably with other studies that achieve good results for data recorded in a single session, the outcomes in the remaining cases in this study are unsatisfactory. It stems from the fact that the number of samples for the increasing number of classes, despite the decreasing period from the first eye movement acquisition session, turned out to be insufficient for the proposed approach.

## 6. Conclusions

The paper presents studies on eye-movement-based biometric identification, for the purpose of which two methods were developed. One of them utilized a feature vector constructed from time series representing eye movement dynamics, including velocity, acceleration, and jerk, as well as their point-to-point percentage changes. The representation in the frequency domain was also taken into account. All time series were calculated for three 100-element segments extracted from the eye movement signal. The LSTM model was used in this case. The second approach relied on statistical descriptors derived from selected time series and utilized a dense (fully connected) neural network. Both models were trained and validated using data registered for users who observed a jumping point on the screen. Very good results were obtained, especially for the LSTM model and the time-series-based feature vector set, with an accuracy of 96%. The dense neural network, based on statistical features, reached 74% accuracy.

Although the research yielded satisfactory results for relatively simple methods, further improvements are planned to enhance eye-movement-based identification. They will include verification of the proposed methods using various stimulus types and various eye movement datasets. Moreover, a fusion of solutions at both the process and results levels will be considered. Different versions of cross-validation that take into account aging visual patterns will also be investigated.

## Figures and Tables

**Table 1:** The description of each session in the GazeBase dataset.

Session	Users	Date from	Date to
1	322	September 2013	February 2014
2	136	February 2014	March 2014
3	105	March 2014	April 2014
4	101	April 2014	April 2014
5	78	September 2014	November 2014
6	59	March 2015	May 2015
7	35	October 2015	November 2015
8	31	March 2016	May 2016
9	14	October 2016	November 2016

**Table 2:** Eye movement features.

Signal	Formula	Description
velocity (v)	$v=\frac{\partial x}{\partial t}$	the first derivative
acceleration (a)	$a=\frac{\partial v}{\partial t}$	the second derivative
jerk (j)	$j=\frac{\partial a}{\partial t}$	the third derivative
velocity change (vc)	$vc=\frac{v[i+1]-v[i]}{v[i]}$	the percentage change of velocity
acceleration change (ac)	$ac=\frac{a[i+1]-a[i]}{a[i]}$	the percentage change of acceleration
jerk change (jc)	$jc=\frac{j[i+1]-j[i]}{j[i]}$	the percentage change of jerk
Fourier transform (Ft)	$A_{k}=\sum_{m=0}^{n-1}a_{m}exp\left\{-2\pi i\frac{mk}{n}\right\}$	the amplitude spectrum, $A_{k}$, k-th component

**Table 3:** Number of samples for 9-session subsets with varying stimulus numbers.

Sessions *S*	Stimulus Positions *X*	Labels Nb *l*	Total	Training	Testing
9	50	14	18,900	16,800	2100
9	35	14	13,230	11,760	1470
9	25	14	9450	8400	1050

**Table 4:** The results obtained in the experiments with the usage of the time-series-based feature set for three numbers of stimulus positions. The standard deviation for each metric was provided in brackets.

Sessions *S*	Stimulus Positions *X*	Accuracy	F1 Score	Cohen’s Kappa
9	50	0.96 (0.06)	0.95 (0.08)	0.96 (0.06)
9	35	0.90 (0.09)	0.87 (0.11)	0.89 (0.09)
9	25	0.79 (0.10)	0.73 (0.11)	0.77 (0.10)

**Table 5:** Number of labels and samples for various subsets of the GazeData dataset in experiments with varying session number.

Sessions *S*	Stimulus Positions *X*	Labels Nb *l*	Total	Training	Testing
8	50	26	31,200	27,300	3900
7	50	29	30,450	26,100	4350
6	50	52	46,800	39,000	7800
5	50	65	48,750	39,000	9750
4	50	85	51,000	38,250	12,750
3	50	90	40,500	27,000	13,500

**Table 6:** The results obtained in the experiments utilizing the time-series-based feature set and a different number of sessions considered. The standard deviation for each metric was provided in brackets.

Sessions *S*	Accuracy	F1 Score	Cohen’s Kappa
8	0.87 (0.06)	0.83 (0.08)	0.86 (0.06)
7	0.83 (0.05)	0.78 (0.06)	0.82 (0.05)
6	0.73 (0.07)	0.67 (0.09)	0.72 (0.07)
5	0.67 (0.06)	0.60 (0.06)	0.66 (0.06)
4	0.51 (0.02)	0.42 (0.02)	0.50 (0.02)
3	0.34 (0.02)	0.25 (0.01)	0.33 (0.02)

**Table 7:** The results obtained in the experiments with the usage of the statistical feature set and for three numbers of stimulus positions. The standard deviation for each metric was provided in brackets.

Sessions *S*	Stimulus Positions *X*	Accuracy	F1 Score	Cohen’s Kappa
9	50	0.74 (0.08)	0.67 (0.09)	0.72 (0.08)
9	35	0.60 (0.08)	0.50 (0.09)	0.56 (0.09)
9	25	0.55 (0.08)	0.44 (0.09)	0.52 (0.07)

**Table 8:** The results obtained in the experiments using the statistical feature set and varying numbers of sessions considered. The standard deviation for each metric was provided in brackets.

Sessions *S*	Accuracy	F1 Score	Cohen’s Kappa
8	0.49 (0.06)	0.37 (0.05)	0.47 (0.06)
7	0.47 (0.06)	0.37 (0.06)	0.45 (0.06)
6	0.33 (0.02)	0.26 (0.02)	0.31 (0.02)
5	0.25 (0.04)	0.16 (0.02)	0.23 (0.04)
4	0.16 (0.09)	0.09 (0.05)	0.15 (0.09)
3	0.09 (0.06)	0.04 (0.03)	0.08 (0.06)

**Table 9:** The best-achieved results for the comparable research presented in this paper.

References	Sessions Nb	Time Span	Users Nb	Stimulus Type	Metric Type	Metric Value
Holland et al. [39]	4	1 week	32	text	R1 ACC	83%
Cantoni et al. [37]	3	20 days	88	image	AUC	82%
Zhang et al. [9]	1	-	109	random ^1^	ACC	83%
George et al. [11]	2	20 min	153	random	R1 ACC	90%
George et al. [11]	2	20 min	153	text	R1 ACC	92%
George et al. [11]	2	1 year	37	random	R1 ACC	79%
George et al. [11]	2	1 year	37	text	R1 ACC	83%
Bayat et al. [14]	1	-	40	text	ACC	95%
Liao et al. [19]	1	-	39	task based ^2^	ACC	78%
Seha et al. [20]	1	-	55	video	R1 ACC	97%
D’Amelio et al. [21]	1	-	8	image	ACC	94%
Ozdel et al. [22]	1	-	10	task based ^3^	ACC	93%
ours	9	37 months	14	random	ACC	96%
ours	8	32 months	26	random	ACC	87%
ours	7	26 months	29	random	ACC	83%

^1^ Jumping point, ^2^ Way finding for 4 routes, ^3^ Seven activities (talking, reading, problem-solving, watching a video, typing, walking, and cycling).

## Data Availability

The original contributions presented in this study are included in the article. Further inquiries can be directed to the corresponding author.

## References

[1] Harezlak K., Blasiak M., Kasprowski P. (2021). Biometric Identification Based on Eye Movement Dynamic Features. Sensors.

[2] Alexander R.G., Macknik S.L., Martinez-Conde S. (2018). Microsaccade characteristics in neurological and ophthalmic disease. Front. Neurol..

[3] Otero-Millan J., Macknik S.L., Martinez-Conde S. (2014). Fixational eye movements and binocular vision. Front. Integr. Neurosci..

[4] Kasprowski P., Ober J. (2004). Eye movements in biometrics. Biometric Authentication—ECCV 2004 International Workshop, BioAW 2004, Prague, Czech Republic, 15 May 2004.

[5] Maeder A.J., Fookes C.B. A visual attention approach to personal identification. Proceedings of the Eighth Australian and New Zealand Intelligent Information Systems Conference.

[6] Komogortsev O.V., Jayarathna S., Aragon C.R., Mahmoud M. (2010). Biometric identification via an oculomotor plant mathematical model. Proceedings of the 2010 Symposium on Eye-Tracking Research and Applications.

[7] Kasprowski P., Komogortsev O.V., Karpov A. (2012). First eye movement verification and identification competition at BTAS 2012. Proceedings of the 2012 IEEE Fifth International Conference on Biometrics: Theory, Applications and Systems (BTAS).

[8] Bednarik R., Kinnunen T., Mihaila A., Fränti P. (2005). Eye-movements as a biometric. Image Analysis: 14th Scandinavian Conference, SCIA 2005, Joensuu, Finland, 19–22 June 2005, Proceedings 14.

[9] Zhang Y., Juhola M. (2016). On biometrics with eye movements. IEEE J. Biomed. Health Inform..

[10] Galdi C., Nappi M., Riccio D., Wechsler H. (2016). Eye movement analysis for human authentication: A critical survey. Pattern Recognit. Lett..

[11] George A., Routray A. (2016). A score level fusion method for eye movement biometrics. Pattern Recognit. Lett..

[12] Kasprowski P., Harezlak K. (2014). The second eye movements verification and identification competition. Proceedings of the 2014 IEEE International Joint Conference on Biometrics (IJCB).

[13] Komogortsev O.V., Rigas I. (2015). BioEye 2015: Competition on biometrics via eye movements. Proceedings of the 2015 IEEE 7th International Conference on Biometrics: Theory, Applications and Systems (BTAS).

[14] Bayat A., Pomplun M. (2018). Biometric identification through eye-movement patterns. Advances in Human Factors in Simulation and Modeling: Proceedings of the AHFE 2017 International Conference on Human Factors in Simulation and Modeling, The Westin Bonaventure Hotel, Los Angeles, CA, USA, 17–21 July 2017.

[15] Rigas I., Economou G., Fotopoulos S. (2012). Biometric identification based on the eye movements and graph matching techniques. Pattern Recognit. Lett..

[16] Deravi F., Guness S.P. Gaze trajectory as a biometric modality. Proceedings of the BIOSIGNALS Conference.

[17] Kinnunen T., Sedlak F., Bednarik R. (2010). Towards task-independent person authentication using eye movement signals. Proceedings of the 2010 Symposium on Eye-Tracking Research and Applications.

[18] Rigas I., Komogortsev O.V. (2014). Biometric recognition via probabilistic spatial projection of eye movement trajectories in dynamic visual environments. IEEE Trans. Inf. Forensics Secur..

[19] Liao H., Zhao W., Zhang C., Dong W. (2022). Exploring Eye Movement Biometrics in Real-World Activities: A Case Study of Wayfinding. Sensors.

[20] Seha S.N.A., Hatzinakos D., Zandi A.S., Comeau F.J. (2021). Improving eye movement biometrics in low frame rate eye-tracking devices using periocular and eye blinking features. Image Vis. Comput..

[21] D’Amelio A., Patania S., Bursic S., Cuculo V., Boccignone G. (2023). Using Gaze for Behavioural Biometrics. Sensors.

[22] Özdel S., Meyer J., Abdrabou Y., Kasneci E. (2025). User Identification with LFI-Based Eye Movement Data Using Time and Frequency Domain Features. arXiv.

[23] Meyer J., Frank A., Schlebusch T., Kasneci E. (2022). A CNN-based Human Activity Recognition System Combining a Laser Feedback Interferometry Eye Movement Sensor and an IMU for Context-aware Smart Glasses. Proc. ACM Interact. Mob. Wearable Ubiquitous Technol..

[24] Prasse P., Jäger L.A., Makowski S., Feuerpfeil M., Scheffer T. (2020). On the Relationship between Eye Tracking Resolution and Performance of Oculomotoric Biometric Identification. Procedia Comput. Sci..

[25] Makowski S., Prasse P., Reich D.R., Krakowczyk D., Jäger L.A., Scheffer T. (2021). DeepEyedentificationLive: Oculomotoric Biometric Identification and Presentation-Attack Detection Using Deep Neural Networks. IEEE Trans. Biom. Behav. Identity Sci..

[26] Makowski S., Jäger L.A., Prasse P., Scheffer T. Biometric Identification and Presentation-Attack Detection using Micro- and Macro-Movements of the Eyes. Proceedings of the 2020 IEEE International Joint Conference on Biometrics (IJCB).

[27] Lohr D., Komogortsev O.V. (2022). Eye Know You Too: Toward Viable End-to-End Eye Movement Biometrics for User Authentication. IEEE Trans. Inf. Forensics Secur..

[28] Yin J., Sun J., Li J., Liu K. (2022). An Effective Gaze-Based Authentication Method with the Spatiotemporal Feature of Eye Movement. Sensors.

[29] Griffith H., Lohr D., Abdulin E., Komogortsev O. (2021). GazeBase, a large-scale, multi-stimulus, longitudinal eye movement dataset. Sci. Data.

[30] Wang X., Zhao X., Zhang Y. (2021). Deep-learning-based reading eye-movement analysis for aiding biometric recognition. Neurocomputing.

[31] Qin H., Zhu H., Jin X., Song Q., El-Yacoubi M.A., Gao X. (2025). EmMixformer: Mix Transformer for Eye Movement Recognition. IEEE Trans. Instrum. Meas..

[32] Jia S., Koh D.H., Seccia A., Antonenko P., Lamb R., Keil A., Schneps M., Pomplun M. (2018). Biometric Recognition Through Eye Movements Using a Recurrent Neural Network. Proceedings of the 2018 IEEE International Conference on Big Knowledge (ICBK).

[33] Holland C.D., Komogortsev O.V. (2012). Biometric verification via complex eye movements: The effects of environment and stimulus. Proceedings of the 2012 IEEE Fifth International Conference on Biometrics: Theory, Applications and Systems (BTAS).

[34] Tripathi B., Srivastava V., Pathak V. Human recognition based on oculo-motion characteristics. Proceedings of the AFRICON IEEE 2013.

[35] Zhang Y., Juhola M. On biometric verification of a user by means of eye movement data mining. Proceedings of the 2nd International Conference on Advances in Information Mining and Management.

[36] Cuong N.V., Dinh V., Ho L.S.T. (2012). Mel-frequency cepstral coefficients for eye movement identification. Proceedings of the 2012 IEEE 24th International Conference on Tools with Artificial Intelligence.

[37] Cantoni V., Galdi C., Nappi M., Porta M., Riccio D. (2015). GANT: Gaze Analysis Technique for Human Identification. Pattern Recognit..

[38] Kingma D.P., Ba J. Adam: A Method for Stochastic Optimization. Proceedings of the 3rd International Conference on Learning Representations, ICLR 2015.

[39] Holland C.D., Komogortsev O.V. (2013). Complex eye movement pattern biometrics: Analyzing fixations and saccades. Proceedings of the 2013 International Conference on Biometrics (ICB).

